# Microstructure Optimization of Na_3_SbS_4_/Na_3_Zr_2_Si_2_PO_12_ Composite Solid Electrolytes for Improving Cycling Stability in All‐Solid‐State Sodium Batteries

**DOI:** 10.1002/advs.75364

**Published:** 2026-04-16

**Authors:** Celastin Bebina Thairiyarayar, Zhenghui Pan, Soorathep Kheawhom, Jeng‐Kuei Chang, Wei‐Ren Liu

**Affiliations:** ^1^ Department of Chemical Engineering R&D Center for Membrane Technology Chung Yuan Christian University Taoyuan Taiwan (ROC); ^2^ School of Materials Science and Engineering Tongji University Shanghai China; ^3^ Department of Chemical Engineering Faculty of Engineering Chulalongkorn University Bangkok Thailand; ^4^ Department of Materials Science and Engineering National Yang Ming Chiao Tung University Hsinchu Taiwan (ROC); ^5^ College of Sustainability National Tsing Hua University Hsinchu Taiwan (ROC)

**Keywords:** all‐solid‐state sodium battery, composite solid electrolytes, interfacial stability, Na_3_SbS_4_, NASICON

## Abstract

Sulfide‐based solid electrolytes have attracted significant attention for all‐solid‐state batteries due to their high ionic conductivity. However, their practical application is limited by interfacial instability at the sodium metal anode, leading to side reactions that form Na_2_S and Na_3_Sb, and by structural defects such as voids and cracks that create electronic leakage pathways. To address these issues, a composite electrolyte was developed by incorporating Na_3_Zr_2_Si_2_PO_12_ (NZSP), a stable NASICON‐type oxide, into Na_3_SbS_4_ (NSS). The optimized 90–10 wt.% NSS‐NZSP composite improves microstructural integrity by filling voids and mitigating crack formation, enabling efficient Na^+^ transport. As a result, the ionic conductivity increases from 3.7 × 10^−4^ to 3.97 × 10^−4^ S cm^−1^, while the activation energy decreases from 0.25 to 0.22 eV. A half‐cell configuration (Na_2/3_Fe_1/2_Mn_1/2_O_2_|90–10 wt.% electrolyte|Na) demonstrates stable cycling over 100 cycles at 0.05 A g^−1^, delivering a discharge capacity of 118.9 mAh g^−1^ at room temperature.

## Graphical abstract

This research demonstrates that incorporating 10 wt.% NASICON‐type Na_3_Zr_2_Si_2_PO_12_ into Na_3_SbS_4_ sulfide electrolytes mitigates interfacial instability and structural defects. The resulting composite enhances ionic conductivity to 3.97 * 10^−4^ S cm^−1^ and reduces activation energy. This optimization ensures stable cycling and improved capacity in all‐solid‐state sodium‐metal batteries by suppressing electronic leakage.

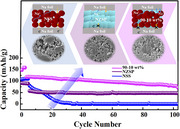

## 1 Introduction

All‐solid‐state batteries (ASSBs) achieved safer operation through the substitution of liquid electrolytes with solid alternatives, which minimized explosion and fire risks stemming from leaks and evaporation [1]. The ionic conductivity of SEs, particularly sulfide‐based types, reaches between 10^−4^ and 10^−2^ S/cm, together with electrochemical stability windows wider than 5 V and low electronic conductivity [2]. The combination of these features enables the attainment of higher energy density with stable long‐term cycling performance [1]. Na_3_SbS_4_ (NSS) has demonstrated better air stability than Na_3_PS_4_ (NPS) and other sulfide‐based SEs. In our previous work [3], we developed an H_2_S sensing system to evaluate moisture sensitivity, revealing that NSS does not emit toxic gases under high‐humidity conditions, whereas NPS does [4]. The ionic conductivity properties of NSS remain unchanged after heating the “air‐exposed” portion of the NSS solid electrolyte, which positions it as a promising material for sodium‐ion battery implementation [5]. The direct use of NSS as a solid‐state sodium battery component faced multiple implementation barriers. The main drawback of NSS was its strong chemical reactivity with metallic sodium, which led to unwanted side reactions that generated Na_2_S and Na_3_Sb products. The reactions in Na||Na cells produced Na_3_Sb and Sb that damaged NSS and generated ion‐blocking layers, which led to battery failure [6, 7, 8]. Several methods existed to overcome these problems. For example, the blockage of unwanted reactions occurred through artificial interphase formation [9] while Na‐Sn alloy anodes replaced pure sodium to decrease reactivity [10] and stability enhancements resulted from halide dopants in sulfide SEs [11] and NSS microstructure modifications improved contact while preventing cracks [12]. Furthermore, adding interfacial layers such as Na_3.4_Zr_2_Si_2.4_P_0.6_O_12_ (NZSPO) between sodium metal and the NSS method established interfaces that either maintain stability naturally or function as beneficial barriers for sodium ion transmission while blocking electron movement [13].

However, the usages of composite solid electrolytes have emerged as a preferred solution because they enhance ionic conductivity while protecting against interfacial damage and reinforcing the material structure [14]. Composite electrolytes achieved their function by sealing the electrolyte's gaps and cracks, which transformed their structure while enabling more paths for ionic movement [15]. Different types of composite SEs have been studied [16]. The improvement of mechanical strength and heat resistance in polymer‐ceramic composites results from adding Al_2_O_3_, TiO_2_, and metal‐organic frameworks (MOFs) fillers to the system [17]. Xu et al. in 2024 demonstrated that the addition of 0.2 wt.% PTFE fibers to NPS resulted in electrolyte strengthening with reduced dendrite growth, but it decreased the NPS ionic conductivity from 1.49 × 10^−4^ to 1.20 × 10^−4^ S/cm [18]. In the case of the NSS solid electrolyte, the addition of PTFE into NSS increased the membrane ionic conductivity to 0.19 mS cm^−1^ due to reduced resistance compared to the pellet form [19].

Apart from inorganic‐based ceramics, polymers have also been combined with NSS to improve its properties. Dong et al. (2024) incorporated carboxymethyl cellulose (CMC) with NSS, resulting in enhanced structural integrity of the composite material. The electrolyte containing 7.5 wt.% NSS‐CMC exhibited improved ionic conductivity, moisture resistance, and electrochemical stability, leading to enhanced cycling performance. The CMC‐modified NSS composite SEs effectively protected NSS from exposure to negative potentials, thereby preventing irreversible reduction [20]. In another study, polyethylene oxide (PEO) was directly applied onto NSS. Although PEO itself does not conduct Na^+^, it acted as a dead‐end pore blocker while NSS preserved its sodium‐ion conductivity. This integration resulted in improved mechanical properties and enhanced interfacial stability [21].

Ceramic‐ceramic composites represented another feasible solution. The doped NSS systems NaI‐NSS [22] and NPSO‐NSS [23], and NaF‐NSS [24] belong to this group in addition to ceramic mixtures. In 2018, Noi's group created a sintering‐free composite by uniting NASICON‐type NZSP (Na_3_Zr_2_Si_2_PO_12_) with NPS that exploited the high conductivity of NZSP and the good interface characteristics of NPS. The dense packing of NZSP‐NPS ball‐milled composites reached 1.1 × 10^−3^ S/cm (at 100°C) conductivity and Na_15_Sn_4_ anodes combined with TiS_2_ cathodes in half cells demonstrated stable cycling over 3 cycles by achieving high reversible capacities around 120 mAh/g at 60 µA cm^−2^ tested at 100°C, which validated the successful operation of oxide‐sulfide composites without requiring high‐temperature sintering for synthesis [25].

NZSP SEs remained the preferred choice because their NASICON‐type structure enables three‐dimensional Na^+^ transport through interconnected SiO_4_ tetrahedra, PO_4_ groups, and ZrO_6_ octahedra. Na^+^ transport occurs through triangular bottlenecks in rhombohedral and monoclinic forms, while these bottlenecks determine the energy barrier and speed of transport [26]. The replacement of Zr^4+^ with aliovalent ions results in better conductivity because it prevents ZrO_2_ formation, but excessive doping causes both contamination and decreased system performance [27]. The addition of NaSiO_3_ promoted grain boundary densification and created Si‐rich secondary phases, which enhanced ionic conductivity levels [28]. NZSP‐Na_3_PO_4_ composites maintained 93% capacity through 550 cycles at 0.5C when used with Na_3_V_2_(PO_4_)_3_ cathodes in half cells, which demonstrated how NZSP enhanced interface stability and ionic transport [29]. In 2023, Goodwin et al. used NZSP as a protective interlayer to separate sulfide electrolytes from reactive anode materials in their experiments. The addition of a dense NZSPO disk between NSS and sodium metal resulted in significant impedance reduction and minimized decomposition. NZSPO exhibits stability against pure sodium as well as Na‐Sn alloys, which prevented reduction at low voltages while protecting the sulfide electrolyte throughout the cycling process [13].

In this study, we extended the concept by integrating NZSP directly into the bulk NSS to form a composite SE, through a simple physical hand mixing without using ball milling and sintering process, and also an electrochemical test was performed at RT. The tetragonal‐phase NSS sample from our laboratory exhibited an ionic conductivity of 3.7 × 10^−4^ S/cm. We enhanced the cycling stability and structural density of NSS by incorporating NZSP in weights ranging from 10% to 30% to 50%. The addition of this composite should both eliminate space between NSS particles and create more efficient sodium ion pathways while decreasing interfacial resistance to produce more stable and efficient all‐solid‐state sodium batteries.

## 2 Experimental Section

### 2.1 Synthesis of Na_3_SbS_4_ (NSS) SEs

**FIGURE 1:**
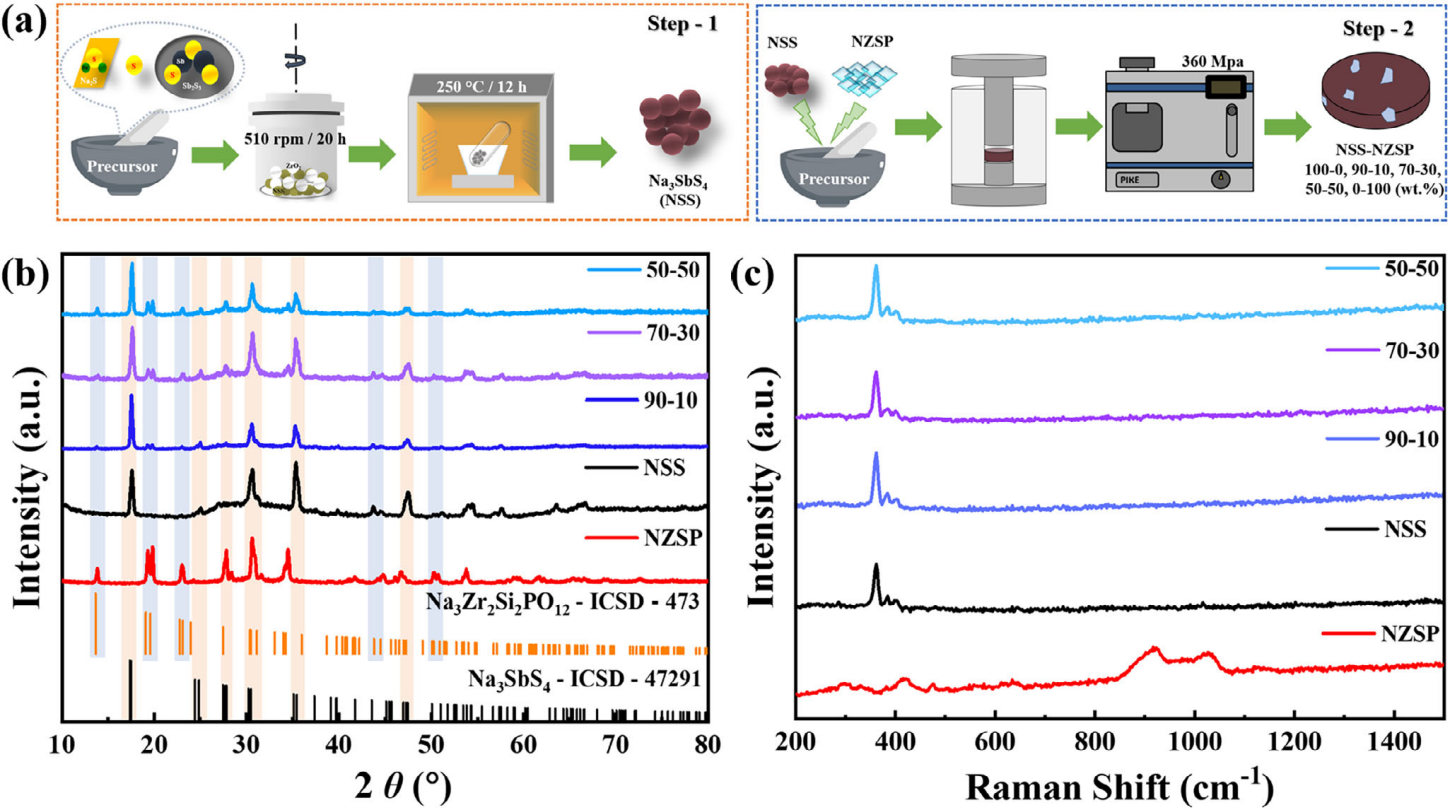
(a) Schematic of the synthesis process for the Na_3_SbS_4_ (NSS) electrolyte and the NSS‐NZSP composites; (b) XRD patterns and (c) Raman spectra of the composites with varying weight ratios of NSS‐NZSP.

Na_3_SbS_4_ (NSS) SEs were synthesized via a high‐energy ball milling approach followed by a sintering process, as described in our previous report [3]. Sodium sulfide (Na_2_S, Alfa Aesar, 90%), antimony(III) sulfide (Sb_2_S_3_, Alfa Aesar, 98%), and sulfur (S, Sigma‐Aldrich, 99.9%) were used as starting materials. These precursors were first manually pregrounded in a 3:1:2 molar ratio using an agate mortar for 10 min, then transferred to a zirconia (ZrO_2_) milling jar with a ball‐to‐powder weight ratio of 10:1. The mixture was milled at 510 rpm for 20 h. Subsequently, the powder was sintered at 250°C for 12 h to obtain the tetragonal phase of Na_3_SbS_4_, referred to as NSS or 100:0 wt.%. Due to the air sensitivity of these samples, all powder handling was carried out inside an argon‐filled glovebox (LABstar, MBraun, München, Germany; H_2_O and O_2_< 0.5 ppm). The overall synthesis procedure is illustrated in Figure 1a as Step‐1.

### 2.2 Preparation of NSS‐NZSP Composite SEs

The preparation of composite SEs involved adding commercial Na_3_Zr_2_Si_2_PO_12_ (NZSP, SKSTC, 99%) to NSS at different weight ratios of 10%, 30%, and 50%, which resulted in compositions denoted as 90–10, 70–30, and 50–50 wt.% (NSS‐NZSP). Pristine NZSP (0:100 wt.%) was used as a reference. The powders received manual mixing in an agate mortar for 10–15 min to achieve uniform blending. The resulting mixtures were pelletized under a uniaxial pressure of 360 MPa using a hydraulic press, as illustrated in Figure 1a, Step 2. All sample handling took place inside the LABstar glovebox (MBraun) to prevent air exposure because the H_2_O and O_2_ levels remained below 0.5 ppm.

### 2.3 Symmetric Sodium Ion Cell Assembly

Symmetric Na|SEs|Na cells were assembled by sandwiching 10 mm diameter composite solid electrolyte pellets between 8 mm diameter Na metal foils (Alfa Aesar) on both sides. Stainless steel spacers and springs were used inside CR2032‐type coin cell casings, and the entire cell was compressed under 210 MPa to ensure good contact. Assembly was performed entirely inside the glovebox to prevent oxidation and moisture‐induced degradation of both solid and composite electrolytes.

### 2.4 Asymmetric Half‐Cell Fabrication

Asymmetric half‐cells were assembled using Na_2/3_Fe_1/2_Mn_1/2_O_2_(NFMO, SKSTC) as the cathode material. A slurry composed of NFMO, PVDF binder, and Super P conductive carbon in an 8:1:1 weight ratio was prepared in N‐methyl‐2‐pyrrolidone (NMP) and stirred overnight to ensure homogeneity. The slurry was cast onto aluminum foil, vacuum‐dried at 120°C for 9 h, and then punched into 10 mm diameter electrodes. Na metal foil was used as the anode, and composite electrolytes of varying NSS‐NZSP compositions were employed as the separator. The assembled cells were compacted under 210 MPa and handled entirely inside the glovebox (LABstar, MBraun, H_2_O and O_2_< 0.5 ppm).

### 2.5 Characterizations

Crystal structure analysis was performed using X‐ray diffraction (XRD, Bruker D2 Phaser) with Cu Kα radiation (30 kV, 10 mA). Samples were sealed in air‐tight holders, and data were collected in the 2θ range of 10°–80° with a scan rate of 2°/min and a step size of 0.02°. Raman spectra were recorded using a BWTEK BAC102‐532E system. Morphology and elemental composition were examined by field‐emission scanning electron microscopy (FE‐SEM, JEOL JSM‐7600F) coupled with energy‐dispersive X‐ray spectroscopy (EDS, X‐MAX, Oxford Instruments). X‐ray photoelectron spectroscopy (XPS) was performed using a K‐Alpha spectrometer (Thermo Scientific, Waltham, MA, USA) to analyze the chemical valence states. The bulk density of each pellet was measured using ethanol immersion. Pellets were weighed in air and then submerged in ethanol, and the density was calculated considering the ethanol density (0.789 g cm^−3^ at 25°C). The relative density of each composite was obtained by comparing the measured bulk density to the theoretical density, calculated from the weight fractions and known densities of NSS (2.90 g cm^−3^) and NZSP (3.27 g cm^−3^).

### 2.6 Electrochemical Measurements

EIS measurements were performed using a Biologic SP‐200 potentiostat. About 70 mg of each electrolyte was pressed into 10 mm diameter pellets at 360 MPa using a PTFE cell. Impedance spectra were recorded over a frequency range of 7 MHz to 10 mHz with an AC amplitude of 10 mV, using two different node point settings of 6 and 30 points per decade. The critical current density tests (Na||Na) were conducted at a current density of 0.1 mA cm^−2^ with 10 min per charge/discharge steps. The linear sweep voltammetry (LSV) was measured using Na||stainless steel cells at a scan rate of 0.1 mV s^−1^. Contact tests (Na|SEs|Na) were conducted at room temperature. The DC polarization measurements were performed using Na|SEs|Na symmetric cells with a bias of 50 mV. For half‐cell testing (NFMO|SEs|Na), the potential window was set between 2.0 and 3.8 V vs. Na/Na^+^. The galvanostatic charge–discharge (GCD) tests were carried out at room temperature using a Neware multi‐channel battery tester.

## 3 Results and Discussion

### 3.1 Structural and Morphological Evolution of NSS‐NZSP Composites

The structural properties of pristine Na_3_SbS_4_ (NSS), commercial Na_3_Zr_2_Si_2_PO_12_ (NZSP), and their composite solid electrolytes were investigated using XRD and Raman spectroscopy, with the results presented in Figure 1. The XRD patterns in Figure 1b showed that the NSS‐NZSP composites were synthesized successfully at various compositions. The patterns exhibited diffraction peaks corresponding to the standard tetragonal phase of NSS (ICSD 47291), with main peaks at 17.6°, 30.6°, and 35.4°, alongside peaks for the monoclinic phase of NZSP (ICSD 473) at 13.9°, 19.2°, 19.8°, 23.08°, 27.7°, and 34.6°. The addition of 10 wt.% NZSP resulted in the appearance of small NZSP peaks, which progressively grew stronger as the NZSP content increased to 30 and 50 wt.%, all without the formation of any impurity phases. This clear superposition of characteristic peaks from both components indicated their chemical compatibility and coexistence as a physical mixture. Complementing the XRD analysis, the Raman spectra in Figure 1c revealed distinct features of both materials, including the sharp, intense symmetric stretching modes of the [SbS_4_]^3−^ tetrahedron at 410, 389, and 368 cm^−1^ from NSS [30], alongside broader vibrational modes at 960 cm^−1^, 1030 cm^−1^ from the NZSP framework of tetrahedral SiO_4_ and PO_4_ [31, 32]. The consistent sharpness and absence of peak shifts in the NSS signals across all composites further confirmed that its fundamental crystal structure remained intact and unaffected by the incorporation of NZSP and the absence of NZSP a weak Raman activity [32] peaks all over mixed composites revealed that there is no chemical bonding/reaction between NZSP and NSS while it was physical mixing of both.

**FIGURE 2:**
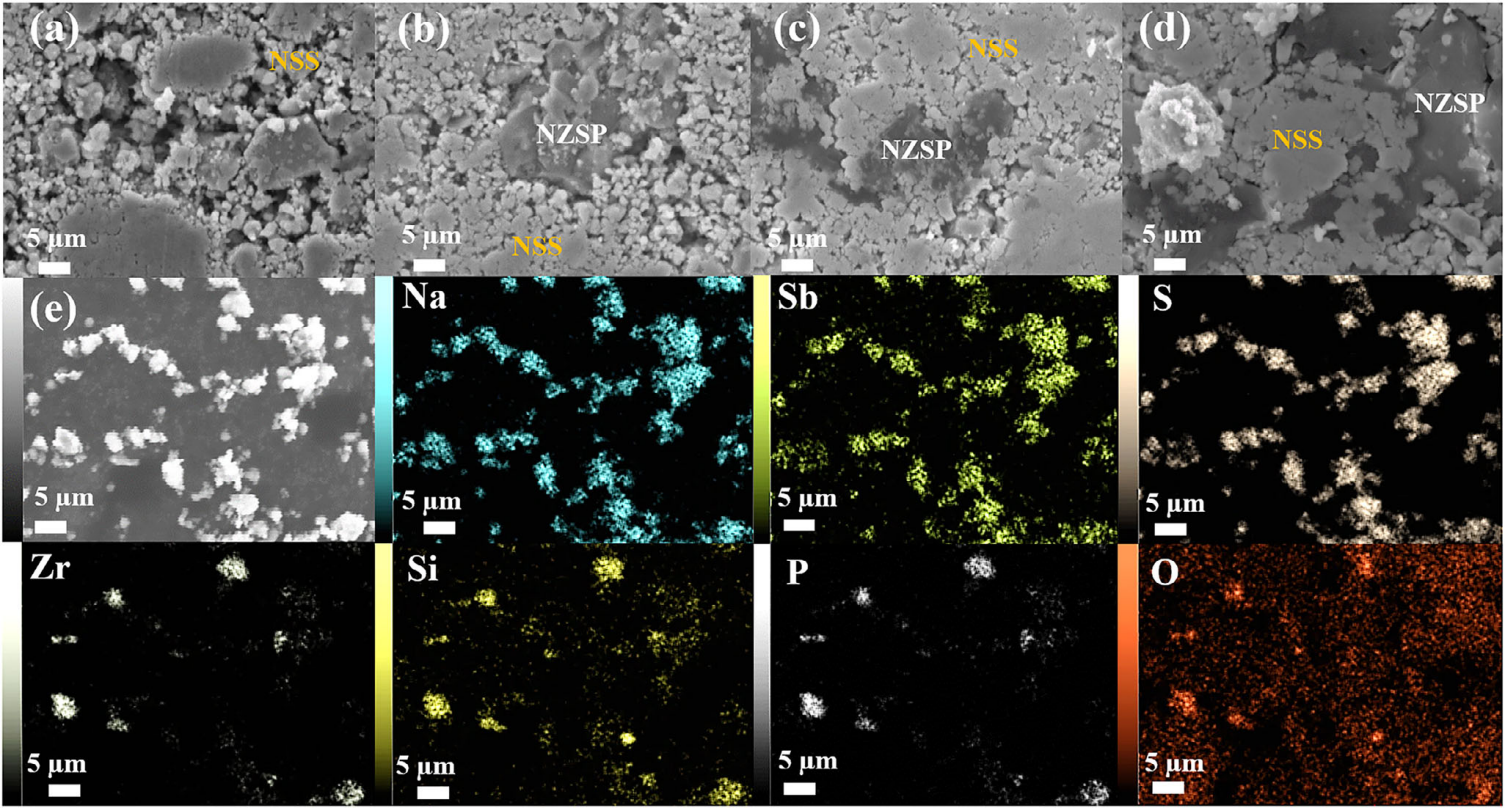
SEM images of pellets: (a) pristine NSS and (b) NSS‐NZSP composites with weight ratios of 90:10, (c) 70:30, and (d) 50:50; (e) SEM image and corresponding EDX elemental mappings of the 90:10 NSS‐NZSP powder.

Surface morphology and microstructure of the pristine NSS and NSS/NZSP composite electrolyte pellets were investigated through SEM analysis. The image of the pristine NSS pellet, presented in Figure 2a, showed a porous structure characterized by significant voids and loosely packed grains of varying sizes, which contributed to high grain boundary resistance and poor grain‐to‐grain contact. However, with the introduction of 10 wt.% NZSP, the microstructure achieved a notably denser and more uniform state, as observed in Figure 2b. This improvement occurred because the finer NZSP particles effectively filled the voids between the larger NSS grains, thereby enhancing grain‐to‐grain contact and establishing more continuous ionic pathways. Meanwhile, at higher NZSP contents of 30 wt.% and 50 wt.%, the images in Figure 2c,d revealed that larger, plate‐like NZSP agglomerates (Figure S4a), began to dominate the structure. This agglomeration disrupted the continuity of the primary NSS conduction network. This comprehensive analysis therefore, identified the 90 wt.% NSS‐10 wt.% NZSP composition as having the optimal microstructure, as it reached maximum compactness while maintaining an interconnected NSS framework, which is critical for attaining superior ionic conductivity and electrochemical performance.

To confirm the compositional homogeneity of as‐prepared 90 wt.% NSS‐10 wt.% NZSP composite SEs, SEM coupled with EDX elemental mapping was performed. The SEM image of the selected region and the corresponding EDX elemental distributions of Na, Sb, S, Zr, Si, P, and O are shown in Figure 2e. For comparison, the elemental maps for pristine NSS, NZSP, and for the 70–30 wt.% and 50–50 wt.% composite SEs are also shown in Figures S1–S3a,b, respectively. The EDX mapping for the optimized composite shown in Figure 2e revealed a uniform distribution of sodium, antimony, and sulfur, originating from the NSS phase, alongside zirconium, silicon, oxygen, sodium, and phosphorus from the NZSP phase. This evidence of intimate mixing confirmed the formation of a homogeneous composite at the microscale. Crucially, this high degree of dispersion created a dense network of stable hetero‐interfaces between the NSS and NZSP particles, which was essential for establishing the continuous, dual‐phase pathways required for enhanced ionic transport.

### 3.2 Enhancement of Ionic Conductivity and Electrochemical Stability of the Composites

**FIGURE 3:**
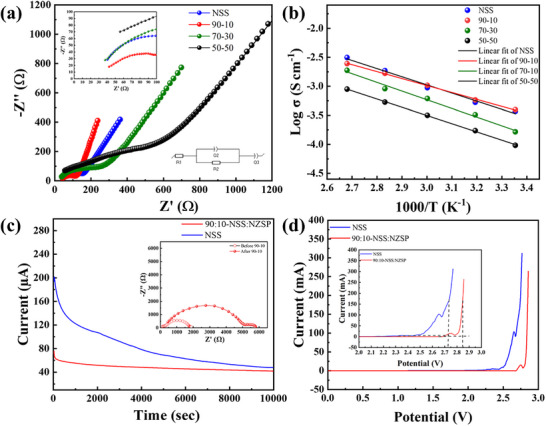
(a) Nyquist plot (Inset: high‐frequency region and equivalent circuit); (b) Arrhenius plots of different wt.% of NZSP in NSS; (c) DC polarization at 0.5 V (Inset: Nyquist plot); (d) LSV (Inset: Expanded view) of NSS and 90–10 wt.% of NSS‐NZSP pellets at scan rate of 0.1 mV s^−1^.

The ionic transport properties of pristine NSS, NZSP, and their composites were evaluated using room‐temperature EIS, as shown in Figure 3. All spectra were acquired with a data density of 30 points per decade to ensure sufficient resolution in the high‐frequency region. As shown in the inset of Figure 3a, the high‐frequency intercept on the Z′‐axis corresponds to the overall resistive response. The resistance components R_1_ and R_2_ were extracted using equivalent circuit fitting, since the Nyquist plots exhibit a single semicircle and do not allow direct separation of bulk and grain boundary contributions. Refinement of the fitting results, as presented in Table 1, indicates that the inclusion of the resistive NZSP phase increases the intrinsic resistance. The 70–30 composite shows a similar high‐frequency intercept to pristine NSS, which is attributed to the reduced pellet thickness of 0.039 cm compared to 0.043 cm for NSS, consistent with the dependence of resistance on the thickness‐to‐area ratio. Despite these variations, the optimized 90–10 composite achieves the lowest total resistance, where R_total_ is defined as the sum of R_1_ and R_2_, reaching 134.6 Ω and yielding a maximum ionic conductivity of 0.397 mS cm^−1^. This represents an improvement over pristine NSS, which shows a conductivity of 0.374 mS cm^−1^. As shown in Table S1, this enhancement is supported by density measurements, where the addition of 10 wt.% NZSP with a uniform particle size of approximately 5 µm (Figure S4a) to the porous NSS framework increases the relative density from approximately 66.2% to 75.2%. This pore‐filling effect reduces high‐resistance solid‐void interfaces and promotes more efficient Na^+^ transport through improved solid‐solid contact. However, increasing the NZSP content to 30% and 50% results in a significant decrease in conductivity to 0.209 and 0.087 mS cm^−1^, respectively. Although the 50–50 composite exhibits a higher relative density of approximately 73.8% compared to 68.4% for the 70–30 sample (Table S1), its ionic conductivity is lower, indicating that densification alone does not govern ionic transport. At moderate NZSP content, such as in the 70–30 composite, a continuous percolation pathway of the highly conductive NSS phase is still maintained, enabling efficient Na^+^ migration. In contrast, at higher NZSP loading, the increasing fraction of the intrinsically resistive NZSP phase disrupts the connectivity of the NSS network, leading to the formation of transport bottlenecks and a more tortuous ion migration pathway. As a result, ionic transport becomes increasingly constrained despite improved pellet densification. To further understand the ion conduction mechanism, temperature‐dependent EIS measurements were performed from 25 to 105°C, as shown in Figure S5b,f. With increasing temperature, the high‐frequency intercept shifts toward lower resistance and the semicircle decreases, indicating enhanced ionic transport due to thermal activation. The Arrhenius plots, shown in Figure 3b and Figure S4b, exhibit linear behavior, confirming a thermally activated conduction process. It should be noted that the activation energy values were derived from EIS data acquired with a data density of 6 points per decade. A comparison between measurements collected at 6 and 30 points per decade is provided in Figure S5a, which demonstrates consistent impedance behavior and negligible deviation in the extracted parameters, confirming the reliability of the activation energy analysis.

The activation energy for Na^+^ transport was calculated from the slope of the Arrhenius plots. Pristine NSS shows an activation energy of 0.25 eV, while the 90–10 composite exhibits a lower value of 0.22 eV. This reduction is attributed to improved interfacial connectivity rather than the intrinsic conductivity of NZSP. In pristine NSS, the relatively low density leads to discontinuous ion pathways due to voids and poorly connected grains. The addition of 10 wt.% NZSP increases the density and enhances particle contact, resulting in a more continuous conduction pathway and reduced energy barriers for Na^+^ migration. In contrast, NZSP exhibits a significantly higher activation energy of 0.46 eV. As shown in Figure S4c,d, the room‐temperature Nyquist plot for NZSP displays a large semicircle of approximately 38 000 Ω, indicating high resistance associated with poor densification. This behavior reflects both the intrinsic transport limitations of the NASICON structure and weak grain boundary connectivity. As the NZSP content increases in the composites, the activation energy rises to 0.29 eV for the 70–30 sample and 0.27 eV for the 50–50 sample, as the resistive NZSP phase increasingly dominates the ion transport pathway. Overall, the 90–10 composition represents the optimal balance, where improved densification and interfacial connectivity enhance ionic transport while preserving the fast conduction pathways of the NSS matrix.

The electrochemical properties of the materials were evaluated using DC polarization and LSV measurements, with the results for each presented in Figure 3c,d. DC polarization tests, presented in Figure 3c, used to assess electronic leakage, revealed that when a constant voltage of 0.5 V was applied, the current for pristine NSS decreased from an initial value of 194 µA to a steady‐state value of 47.8 µA. Meanwhile, the 90–10 composite exhibited a lower overall current under the same 0.5 V bias, which decreased from an initial 75 µA to a steady‐state 41 µA. The corresponding initial and steady‐state resistances, detailed in Figure S6, reached values of 1150 Ω and 4950 Ω for NSS, and 1874 Ω and 5770 Ω for the 90–10 composite (Insets in Figure 3c), respectively. Based on these measurements, the sodium ion transference number was calculated using the Bruce–Vincent equation, $t_{Na^{+}}=\frac{I_{s}\;\left(\Delta V-I_{0}R_{0}\right)}{I_{o}\;\left(\Delta V-I_{s}R_{s}\right)}$ [33]. This calculation revealed a substantial improvement from a $t_{Na^{+}}$ number of 0.24 for pristine NSS to an achieved value of 0.74 for the 90–10 composition. These results confirmed that the composite provided superior electronic insulation and better selectivity for Na^+^ ions, which was critical for suppressing sodium dendrite growth and maintaining interface stability. Furthermore, the electrochemical stability window was investigated using the LSV by using Na|Stainless steel as shown in Figure 3d. The 90–10 composite demonstrated a wider electrochemical stability window, as its oxidative decomposition during the 0.1 mV s^−1^ scan was shifted anodically to approximately 2.85 V, compared to the approximate 2.73 V [34] observed for NSS. This improvement was attributed to the inherently high stability of the NZSP component, which showed stability up to 7 V in the data from Figure S7, indicating that the composite possessed enhanced oxidative resistance, making it more compatible with high‐voltage cathode materials.

**TABLE 1:** Comparison of ionic conductivity, activation energy of NSS, NZSP, and NSS/NZSP composite SEs at different wt.% at RT.

Samples	R_total_ = R_1_ + R_2_ (Ω)	σ_25°C_ (mS cm^−1^)		E_a_ (eV)
NSS	146.2	0.374		0.25
90–10	134.6	0.397	0.22	
70–30	226.7	0.208		0.29
50–50	536.4	0.088		0.27
NZSP	38305	0.0040		0.46

### 3.3 Interfacial Stability and Na Anode Compatibility of Composite Electrolytes

**FIGURE 4:**
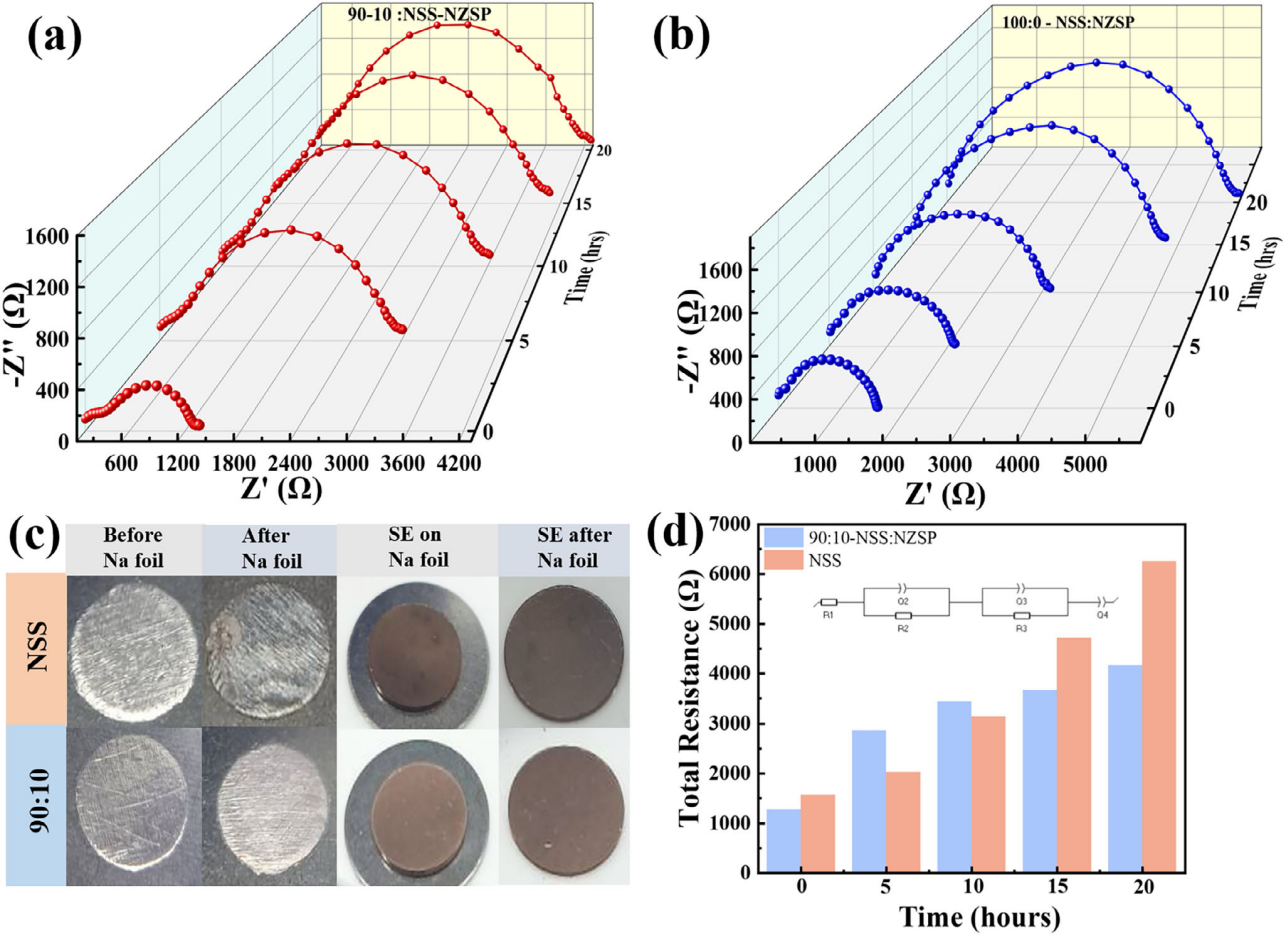
(a, b) Nyquist plots and (c) Photographic images of NSS and 90–10 wt.% NSS‐NZSP pellets before and after 20 h of contact with Na foil; (d) Comparison plot of total resistance as a function of contact time for NSS and 90–10 wt.% NSS‐NZSP pellets at room temperature.

The interfacial stability of pristine NSS and the optimized 90‐10 composite against sodium metal was evaluated by monitoring symmetric cells over 20 h at room temperature, with the results presented in Figure 4. The pristine NSS exhibited poor chemical compatibility, as the data in Figure 4b,d showed its interfacial resistance increased sharply from an initial value of 1569 Ω to 6254 Ω. This instability was visually confirmed by the severe tarnishing observed on the sodium foil's surface, which is displayed in the top image of Figure 4c and indicates an aggressive decomposition reaction at the interface. However, the 90–10 composite demonstrated significantly improved stability. Its interfacial resistance, plotted in Figure 4a,d, increased only moderately from a value of 1272 Ω to 4165 Ω over the same period. Furthermore, the corresponding image in the bottom panel of Figure 4c showed that the sodium foil retained its metallic luster without any signs of degradation. This enhanced stability was attributed to the formation of a well‐structured, passivating solid electrolyte interphase (SEI) that effectively suppressed unwanted side reactions and protected both the electrolyte and the sodium metal. These findings therefore confirmed that incorporating 10 wt.% NZSP was crucial for strengthening the interface against chemical degradation, a key factor in extending electrochemical performance.

**FIGURE 5:**
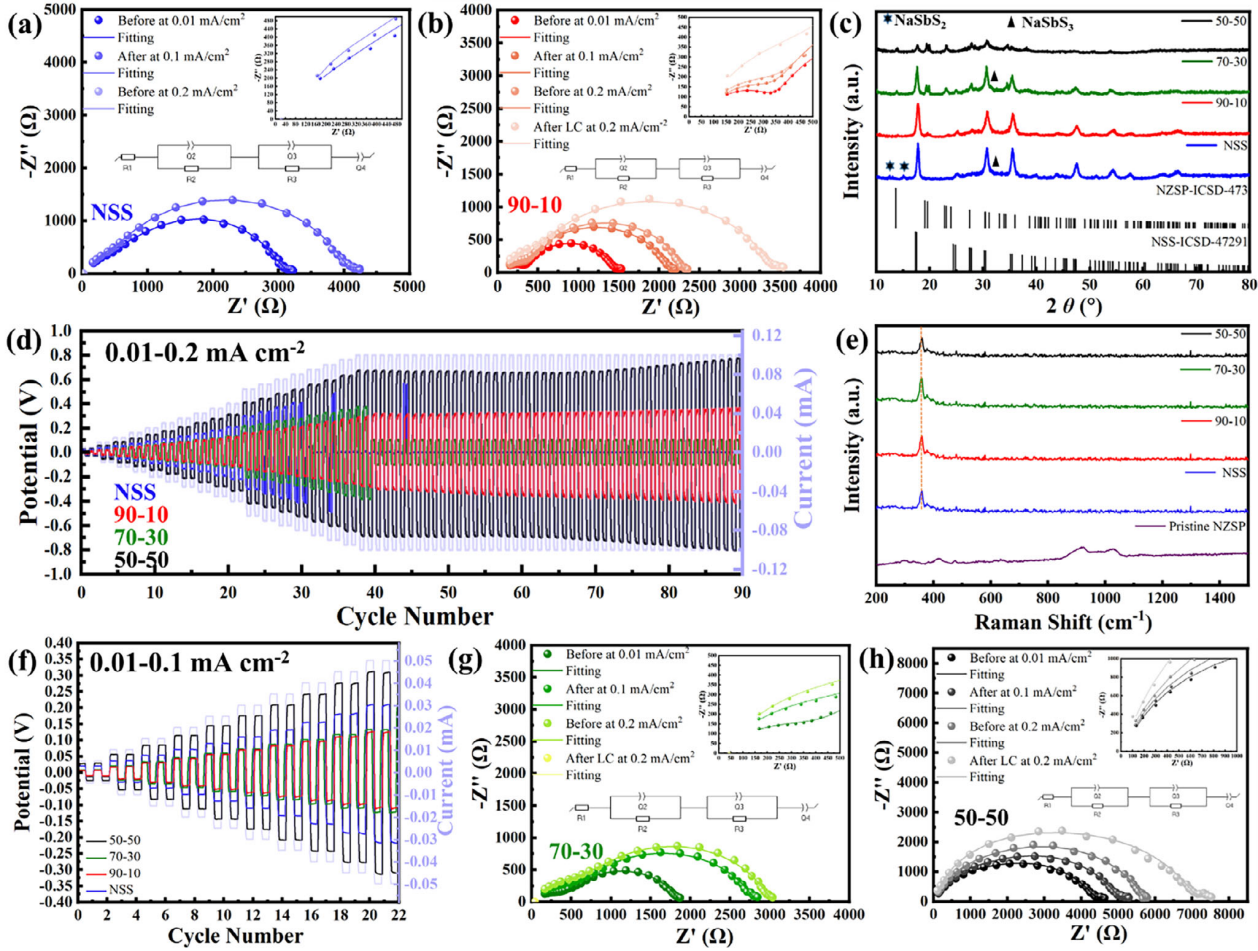
Nyquist plots (Inset: Inset: high‐frequency region and equivalent circuit) of (a) NSS and (b) 90–10, (g) 70–30 and (h) 50–50 wt.% NSS‐NZSP at RT; (c) XRD patterns and (e) Raman spectra of the‐assembled cells after CCD cycled with different composites of NZSP/NSS at RT; (d) CCD profiles at 0.01–0.2 mA cm^−2^, with enlarged views of 0.01–0.1 mA cm^−2^ shown in (d–f) for different ratio of NZSP/NSS by using Na|SEs|Na cells.

Electrochemical stability was assessed using symmetric Na|SEs|Na cells through critical current density tests and long‐term galvanostatic stripping/plating experiments. As observed in Figure 5d, the CCD was evaluated over the range of 0.01–0.2 mA cm^−2^, while a magnified view of the lower current density region (0.01–0.1 mA cm^−2^) is separately shown in Figure 5f. Increasing NZSP content initially reduced the over potential, with 90–10 and 70–30 composites exhibiting lower over potentials, specifically 0.0062 V and 0.007 V respectively, at 0.01 mA cm^−2^ compared to NSS at 0.0184 V and 50‐50 at 0.025 V. Similar, at 0.1 mA cm^−2^, with NSS at 0.2 V, 90–10 at 0.12 V, 70–30 at 0.13 V, and 50–50 at 0.31 V. At a higher current density of 0.2 mA cm^−2^ and after 50 continuous cycles (Figure 5d), the 70–30 composite experienced a short circuit, and NSS exhibited voltage fluctuations, indicating instability. However, the 90–10 composite maintained stable over potential between 0.31 V and 0.36 V, while the 50–50 composite showed significantly higher over potential. This stability difference can be attributed to the changes in resistance values, particularly R_2_ (charge transfer resistance) obtained from the Nyquist plots (Figure 5a,b,g,h) and Table 2. For the 90–10 composite, R_2_ only increased from 264 to 290.6 Ω after cycling, representing a minimal 10.1% increase, whereas for the 50–50 composite, it dramatically increased from 1012 to 1945 Ω, a substantial 92.2% increase, suggesting significantly greater interfacial stability in the 90–10 composite after long‐term cycling (LC). The reason the 90–10 composite exhibits this superior stability was rooted in its ability to maintain a robust and minimally resistive interface, crucial for efficient and stable Na‐ion transport.

**TABLE 2:** Resistance values of NSS, NZSP, and composites from CCD at RT.

Na||Na at RT	Current Density (mA/cm^2^)	R1(Ω)	R2(Ω)	R3(Ω)
NSS	Before–0.01	54.6	510.9	2554
	After–0.1	61.91	280.73	3519
	Before–0.2	1.84	0.013	21.498
90–10	Before–0.01	75.2	264.1	1092.7
	After–0.1	87.35	235.9	1790
	Before–0.2	71	291.8	1942
	After LC–0.2	80.3	290.6	3021
70–30	Before–0.01	84.5	489.5	1361
	After–0.1	88.2	580.3	2214
	Before–0.2	92.6	640	2311
50–50	Before–0.01	98.4	1012	3194
	After–0.1	104.7	1066	3797
	Before–0.2	101.3	874.51	4847
	After LC–0.2	110.8	1945	5052

The XRD patterns, as shown in Figure 5c provide insights into the material's phase composition and the presence of impurities after critical current density (CCD) testing and long cycling. The intensity of NSS peaks was reduced in the composites. Notably, the 90‐10 composite showed no significant impurity peaks even after LC, with the NZSP peaks being almost undetectable, suggesting excellent integration without the formation of new detrimental phases. However, NSS, 70–30, and 50–50 composites revealed the presence of Na_3_SbS_2_ (PDF‐27‐0638 at 15.09° and 31°, and PDF 84 ‐1633 at 12.30°) and Na_3_SbS_3_ (PDF 43–0819 at 17.8°), which were likely decomposition products or impurities that contribute to an increase over potential and reduced stability in those ratios. The absence of these impurity phases in the 90‐10 composite after cycling further underscores its superior chemical stability. Raman spectroscopy (Figure 5e) further supports these findings, specifically from the solid electrolyte pellet after LC. The absence of any discernible peaks corresponding to NZSP in the Raman spectra indicates a lack of chemical bonding or interfacial phase formation between NSS and NZSP. This suggests that the composites are primarily physical mixtures rather than chemically bonded structures. The weak Raman activity of phosphate‐based oxides like NZSP [32] also contributes to their invisibility in the spectra, further reinforcing the idea of a composite where the individual components largely retain their identities without significant interaction that would lead to new vibrational modes. Crucially, the absence of new, detrimental vibrational modes in the 90–10 composite after long cycling, which would correspond to impurities observed in XRD for other ratios, confirms its structural integrity and highlights why its low resistance and low increased charge transfer persist, making it the best performer.

### 3.4 Electrochemical Performance of NFMO Half‐Cells with Composite Electrolytes

**FIGURE 6:**
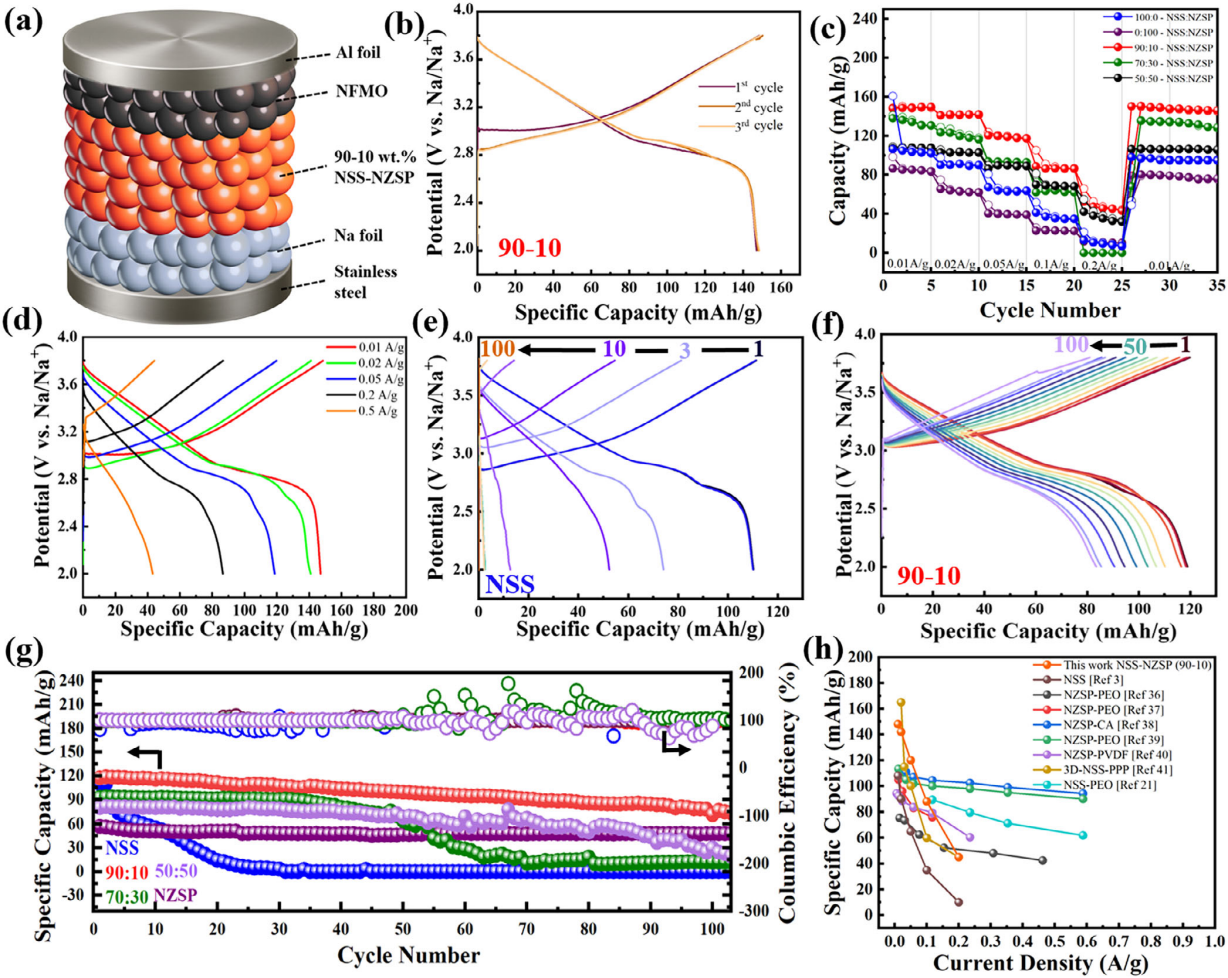
(a) Schematic of the cell configuration of NFMO|NSS‐NZSP|Na at RT; (b) Galvanostatic charge–discharge cycles of the 90:10 cell at the first three at current density of 0.01 A/g; (c) Rate capability comparison of cells with different wt.% of NZSP in NSS; (d) Voltage profiles of the 90:10 cell at various current densities; (g) Cycling performance of different wt.% of NZSP in NSS at 0.05 A/g; (e, f) Representative charge–discharge profiles from the cycling stability tests in (g) for cells using (e) pristine NSS and (f) the 90:10 composite, respectively; (h) Benchmarking of the cycling performance against previously reported all‐solid‐state sodium batteries [3, 21, 36, 37, 38, 39, 40, 41].

To evaluate their electrochemical practicability, NFMO|SEs|Na all‐solid‐state cells were assembled using the various NSS‐NZSP composite electrolytes, as illustrated in the schematic of Figure 6a. The initial galvanostatic charge–discharge profiles of the optimized 90–10 composite at a current density of 0.01 A/g, shown in Figure 6b, confirmed its balanced excellence. It delivered a high first‐cycle reversible discharge capacity of 148.6 mAh/g with an outstanding coulombic efficiency of 99.6%. The rate capability test, shown in Figure 6c, and the corresponding charge–discharge profiles at various rates in Figure 6d, revealed the superior kinetic performance of the 90–10 composite. It achieved average discharge capacities of 148.0, 142.0, 118.9, 86.4, and 47.3 mAh/g at current densities of 0.01, 0.02, 0.05, 0.1, and 0.2 A/g, respectively. For comparison, as shown in Figure S8, all other wt.% showed lower first‐cycle capacities at 0.01 A/g: pristine NSS (Figure S8a) achieved 106 mAh/g [3], the 70–30 composite (Figure S8c) reached 73.1 mAh/g, the 50–50 composite (Figure S8d) delivered 107.2 mAh/g, and commercial NZSP (Figure S8b) showed 86.6 mAh/g. The comparative profiles in Figure S8 also showed a clear trend where the voltage hysteresis progressively decreased as the NZSP content increased. This indicates that the addition of NZSP, a Na^+^ superionic conductor, introduced a dedicated ionic pathway that significantly reduced internal resistance and resulted in flatter, well‐defined voltage plateaus.

Long‐term cycling stability of the electrolytes was evaluated in NFMO|SEs|Na cells at a current density of 0.05 A/g, which revealed dramatic performance differences as detailed in the comparative plot of Figure 6g. Representative charge–discharge profiles taken from this stability test are presented in Figure 6e for the pristine NSS cell and in Figure 6f for the 90–10 composite. The cycling performance in Figure 6g,e shows the pristine NSS cell suffered from severe interfacial instability; it initially showed a capacity of 113.2 mAh/g but rapidly decreased to 75.1 mAh/g in just three cycles. However, the optimized 90–10 composite demonstrated both high capacity and exceptional stability. As shown in Figure 6g,f, it delivered an initial discharge capacity of 118.9 mAh/g and maintained this value with minimal degradation over 100 cycles, all while sustaining a nearly perfect coulombic efficiency above 99.7%. The stability is visually confirmed by the highly overlapping voltage profiles in Figure 6f. The cycling performance in Figure 6g also confirms that 10 wt.% was the optimal amount of NZSP. The 70–30 and 50–50 composites delivered lower initial capacities of 93.3 mAh/g and 81.2 mAh/g, respectively, and began to degrade significantly after only 35 and 50 cycles. Meanwhile, the commercial NZSP cell, while very stable, offered a very low capacity of just 53.8 mAh/g. These results conclusively demonstrated that the 90–10 composite is an ideal balance to achieve unparalleled long‐term stability. Furthermore, this outstanding performance was benchmarked against other previously reported NSS‐based systems in Figure 6h, confirming the state‐of‐the‐art stability and capacity retention achieved in this work.

### 3.5 Interfacial Degradation and Reaction Mechanism During Cycling

**FIGURE 7:**
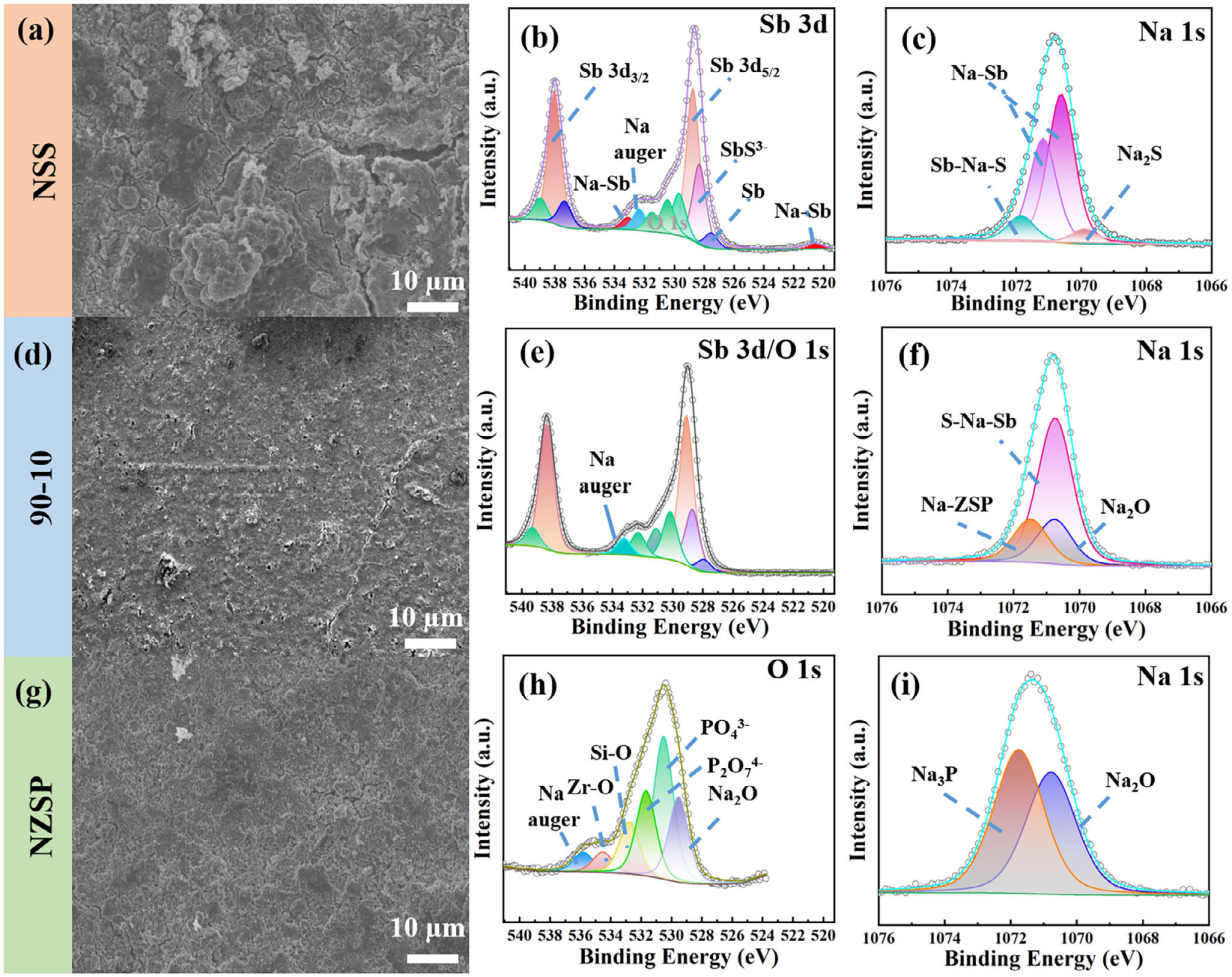
(a–i) Post‐cycling analysis of the electrolyte interfaces: SEM images of the (a) pristine NSS and (d) 90:10 composite and (g) NZSP; Corresponding XPS spectra for the (b)—Sb 3d, c—Na 1s) NSS and (e)‐ Sb 3d/O 1s, f—Na) 90:10 composite and (h)—O 1s, (i)—Na). All post‐cycling tests were performed at RT after cycling at 0.05 A/g.

To investigate the stability of the SEI film and the evolution of cell resistances, EIS was performed before and after 100 cycles, with the results presented in Figure 7 and Figure S9 and summarized in Table S2. The initial state of the cells, detailed in Figure S9a, already revealed significant differences. The pristine NSS cell exhibited high initial resistance, showing a charge transfer resistance (R_2_) of 443.3 Ω and an interfacial resistance (R_3_) that reached 1678 Ω. However, the 90–10 composite demonstrated far superior initial kinetics, with its charge transfer resistance measuring a much lower 66.72 Ω and its initial interfacial resistance showing a value of only 148.8 Ω. This performance gap became catastrophic after 100 cycles of operation (Figure S9b). The interfacial resistance of the pristine NSS cell increased dramatically to a value of 5835 Ω, indicating complete interfacial collapse and serious side reactions. Meanwhile, the interfacial resistance of the highly stable 90–10 composite showed only a controlled increase to a final value of 449 Ω, confirming the formation of a durable and effective passivating SEI. These results proved that the 90–10 composition achieved an ideal equilibrium. NZSP particles fostered a stable interface with low initial and final resistance, which prevented the runaway degradation that plagued pristine NSS and ultimately led to the premature failure observed in the 70–30 and 50–50 cells.

To obtain a systematic understanding of the mechanism behind stabilities at ion conducting interfaces during cycling, post‐mortem characterizations to characterizing Na metal anode with solid electrolyte by SEM and XPS were also performed, and a detailed discussion was included in Figure 7. For the cell using pristine NSS, the SEM image in Figure 7a exposed a heavily deteriorated anode surface with large cracks and fragmented moss growth structure. This directly implied the presence of an immature solid electrolyte interphase (SEI) and unmanaged dendritic sodium growth. XPS analyses confirmed that this physical collapse stemmed from the electrochemical decomposition of NSS. The post‐cycling Sb 3d spectrum was displayed in Figure 7b, a remarkable reduction in the pristine [SbS_4_]^3−^ building block (with Sb 3d_5/2_ at 528.8 eV and 538.0 eV for Sb 3d_3/2_) was observed. Interestingly, new peaks attributed to elemental Sb at 528.0 eV and importantly, an electronically conductive Na‐Sb alloy (Na_3_Sb) at 523.0 eV were observed simultaneously.

These are the reduction forms of SbS_4_^3−^ that were also implied by the XRD data after CCD, showing the formation of metallic Sb or Na‐Sb alloys. The Na 1s spectrum in Figure 7c further corroborated this finding by showing distinct peaks for Na‐Sb bonding, confirming the formation of the alloy, and Na_2_S, with its characteristic peak at 1068.5 eV. The simultaneous formation of electronically conductive Na‐Sb and ionically insulating Na_2_S created a detrimental, mixed‐conducting interphase that accelerated parasitic reactions, ultimately leading to rapid cell failure [9]. In comparison, the Na anode side of the 90–10 composite cell has been stable. The SEM image in Figure 7d exhibited dense, compact, and smooth surface in which no structural degradation or dendritic characteristic was observed. This excellent stability was well demonstrated by XPS spectra in Figure 7e,f.

The Sb 3d spectrum, presented in Figure 7e, showed that the chemical state of the [SbS_4_]^3−^ unit was well‐preserved, as its characteristic peaks for Sb 3d_5/2_ at 528.8 eV and Sb 3d_3/2_ at 538.0 eV remained dominant, indicating significantly reduced NSS decomposition. Importantly, although the O 1s spectra were overlapped by the dominant Sb 3d signals in the 90–10 composite, the persistence of the Sb 3d signal as dominant further confirms the stability of the [SbS_4_]^3−^ unit. Crucially, no electronically conductive Na‐Sb alloy was detected, eliminating a major pathway for parasitic reactions. Similarly, the Na 1s spectrum shown in Figure 7f indicated that the primary Sb─S─Na bridging bonds were largely retained, and it clearly shows Na signals associated with both NSS and NZSP. This 90–10 composite significantly mitigates the severe side reactions observed in NSS by reducing the SbS_4_^3−^ unit reduction and inhibiting the formation of both electronically conductive elemental Sb/Na‐Sb alloy and ionically insulating Na_2_S. This is achieved by forming a stable SEI, particularly with the generation of Na_2_O, which is prominently observed as a peak in the Na 1s spectrum at 1070.0 eV, along with a peak for Na‐ZSP bonding. This Na_2_O originates from the unreacted NZSP, and as demonstrated by Figure 7g‐i, NZSP itself is a stable component. Figure 7g shows the SEM image of pristine NZSP, while Figure 7h displays its O 1s spectrum, revealing distinct peaks for Si‐O, PO_4_^3−^, Na‐Zr‐O, and Na_2_O, confirming its multi‐component nature and stability. The Na 1s spectrum for NZSP (Figure 7i) clearly shows a Na_3_P peak at 1072.0 eV and a Na_2_O peak at 1070.0 eV, indicating the presence of Na_2_O within NZSP itself. This inherent stability of NZSP, coupled with its ability to form Na_2_O as a stable interface [35], is crucial in reducing the side reactions and promoting a stable, ionically conductive SEI that is able to endure very stable long‐term cycling.

**FIGURE 8:**
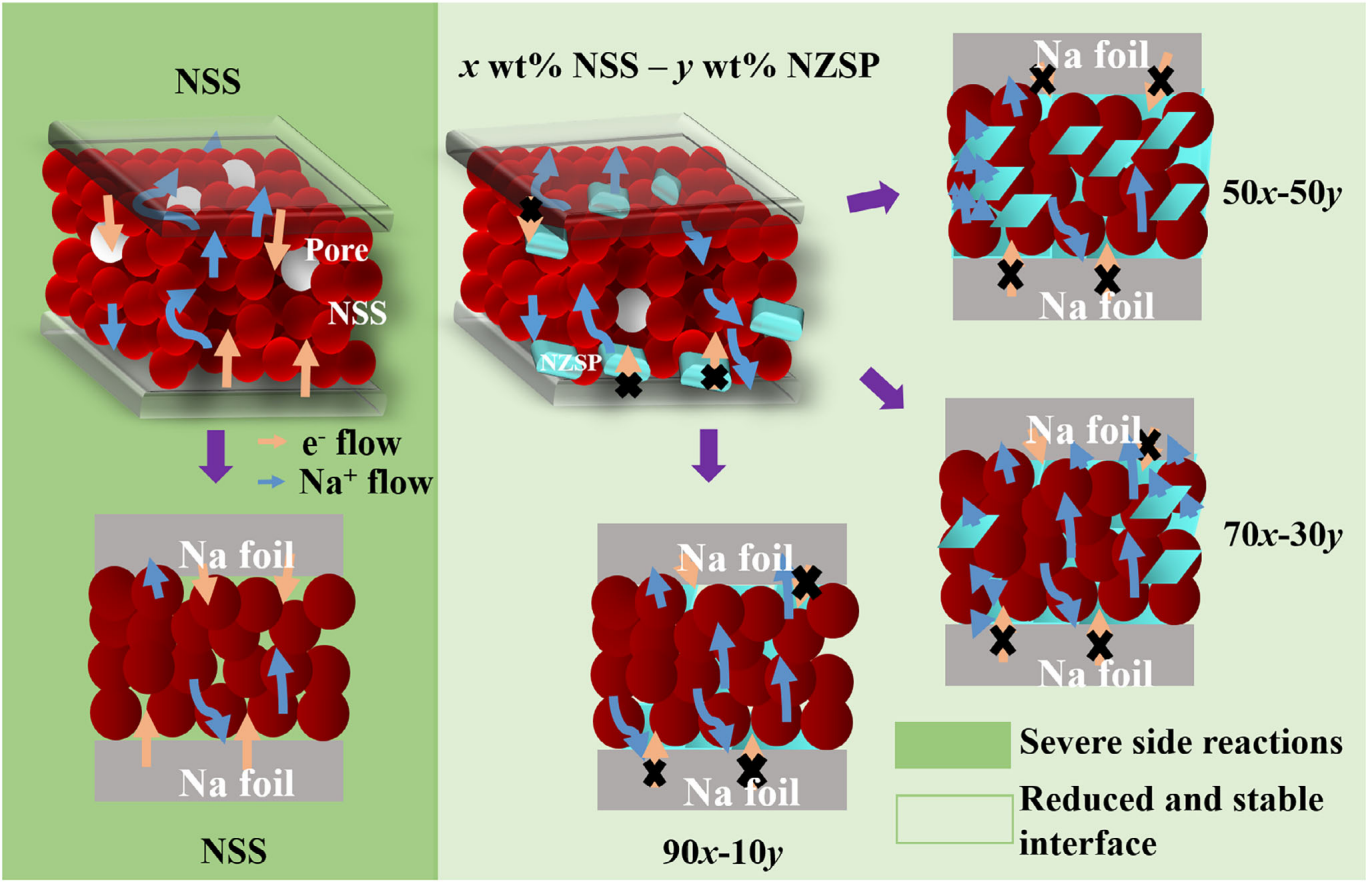
Schematic of Na interface stabilization through NZSP addition (light green) to NSS (dark green), forming a stable interface after 100 cycles in an NFMO|SEs|Na cell at 0.05 A/g.

The origin of enhanced interfacial stability in NSS‐NZSP composites is illustrated in Figure 8. Direct contact between pristine NSS and Na metal induces electrochemical decomposition, forming unstable interphase products. Specifically, Na_3_SbS_4_ undergoes reductive decomposition via Sb(V) reduction and sulfide framework collapse: Na_3_SbS_4_ (s) + *x* Na (s) → Na_3_Sb (s) + Na_2_S (s), Na_3_SbS_4_ + 2Na → Na_3_SbS_3_ + Na_2_S [9]. This reaction produces a mixed‐conducting interphase (MCI) consisting of electronically conductive Na‐Sb alloy and ionically conductive but electronically insulating Na_2_S. The presence of electronically conductive Na–Sb species enables continuous electron transport across the interface, sustaining electrolyte decomposition, promoting dendrite formation, and ultimately leading to rapid cell failure. Incorporation of NZSP modifies this interfacial chemistry in a composition‐dependent manner. At the optimal 90:10 NSS:NZSP ratio, finely dispersed NZSP particles act as structural fillers, increasing relative density (∼75%) and homogenizing the interface. Rather than forming a continuous coating, NZSP locally decorates the NSS surface and introduces Na_2_O as a key SEI component. Together, Na_2_S and Na_2_O form a composite SEI comprising Na^+^‐conductive yet electronically insulating phases. In particular, Na_2_O plays a critical role in disrupting electron percolation pathways associated with Na‐Sb species, thereby stabilizing the interface. This results in a “balanced” SEI that permits Na^+^ transport while suppressing further electrolyte reduction. Importantly, the effectiveness of this SEI depends on controlled phase distribution. While both Na_2_S and Na_2_O are ionically conductive, excessive accumulation, particularly of Na_2_O with its relatively lower ionic conductivity, can increase interfacial resistance and hinder Na^+^ transport. At higher NZSP fractions (70:30 and 50:50), interfacial stability is further enhanced, but at the expense of transport. The low intrinsic conductivity of cold‐pressed NZSP (0.0040 mS cm^−1^ at 360 MPa), combined with the buildup of insulating interphase products, introduces a transport bottleneck that limits Na^+^ flux and obstructs the high‐conductivity pathways of the NSS matrix. Thus, the 90:10 composition represents an optimal balance between interfacial stability and ionic transport. This “sweet spot” suppresses decomposition while maintaining efficient Na^+^ conduction, resulting in improved cycling stability and overall electrochemical performance.

## 4 Conclusions

In this study, we firstly demonstrate a successful composite electrolyte strategy to overcome the critical limitations of poor interfacial stability and physical non‐uniformity for pristine Na_3_SbS_4_ (NSS). Through the mechanical mixing process, 10 wt.% of the superionic conductor Na_3_Zr_2_Si_2_PO_12_ (NZSP) was incorporated into the matrix without disrupting the basic NSS crystal structure. This process effectively filled voids within SEs, resulting in improved compaction and the formation of a synergistic, biphasic Na^+^ conduction network. The structural enhancement increased the total ionic conductivity to 3.97 × 10^−4^ S cm^−1^ and reduced the activation energy to 0.22 eV. More significantly, the incorporation of NZSP leveraged its wide electrochemical stability window to engineer a robust and stable interface with the sodium metal anode, effectively passivating the reactive NSS surface and mitigating the detrimental side reactions that typically cause rapid cell failure. The success of this approach was validated by the excellent performance of the resulting NFMO|90–10 wt.%|Na half‐cell, which delivered a high initial specific capacity of 148 mAh g^−1^ at a current density of 0.01 A g^−1^, and demonstrated outstanding cycling stability for 100 cycles at 0.05 A g^−1^ at room temperature. These findings highlight that a rationally designed composite approach is a highly effective pathway for developing practical and long‐lasting sulfide‐based all‐solid‐state sodium batteries.

## Conflicts of Interest

The authors declare no conflicts of interest.

## Supporting information




**Supporting File**: advs75364‐sup‐0001‐SuppMat.pdf.

## Data Availability

The authors have nothing to report.
