# Supplementary material for: Microstructure Optimization of Na3SbS4/Na3Zr2Si2PO12 Composite Solid Electrolytes for Improving Cycling Stability in All‐Solid‐State Sodium Batteries
Source: Adv Sci (Weinh). 2026 Apr 16;13(39):e75364. doi: 10.1002/advs.75364 (PMC13335589; doi:10.1002/advs.75364)
Supplement: Supplementary file 1 — Supporting File: advs75364‐sup‐0001‐SuppMat.pdf. [file ADVS-13-e75364-s001.pdf]

Supporting Information

Microstructure optimization of $Na_3SbS_4/Na_3Zr_2Si_2PO_{12}$ composite solid electrolytes for improving cycling stability in all-solid-state sodium batteries

Celastin Bebina Thairiyarayar,[a] Zhenghui Pan,[b] Soorathep Kheawhom,[c] Jeng-Kuei Chang[d] and Wei-Ren Liu[a,c,e,*]

[a] *Department of Chemical Engineering, R&D Center for Membrane Technology, Chung Yuan Christian University, No. 200 Chung Pei Road, Chungli District, Taoyuan City, 32023, Taiwan, ROC*

[b] *School of Materials Science and Engineering, Tongji University, Shanghai 201804, China*

[c] *Department of Chemical Engineering, Faculty of Engineering, Chulalongkorn University, Bangkok, 10330, Thailand*

[d] *Department of Materials Science and Engineering, National Yang Ming Chiao Tung University, Hsinchu 30010, Taiwan*

[e] *College of Sustainability, National Tsing Hua University, No. 101, Section 2, Guangfu Road, Hsinchu City, 30013, Taiwan, R.O.C.*

**E-mail address: WRLiu1203@gmail.com*

Tel: +886 3-265-4140

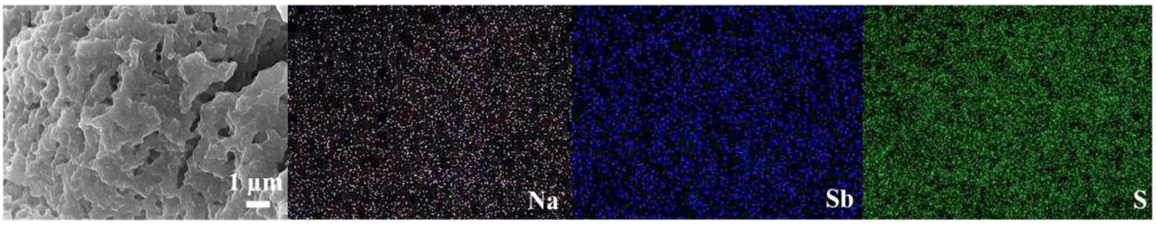

Fig. S1. SEM/EDX mapping of Na_3SbS_4 powder.

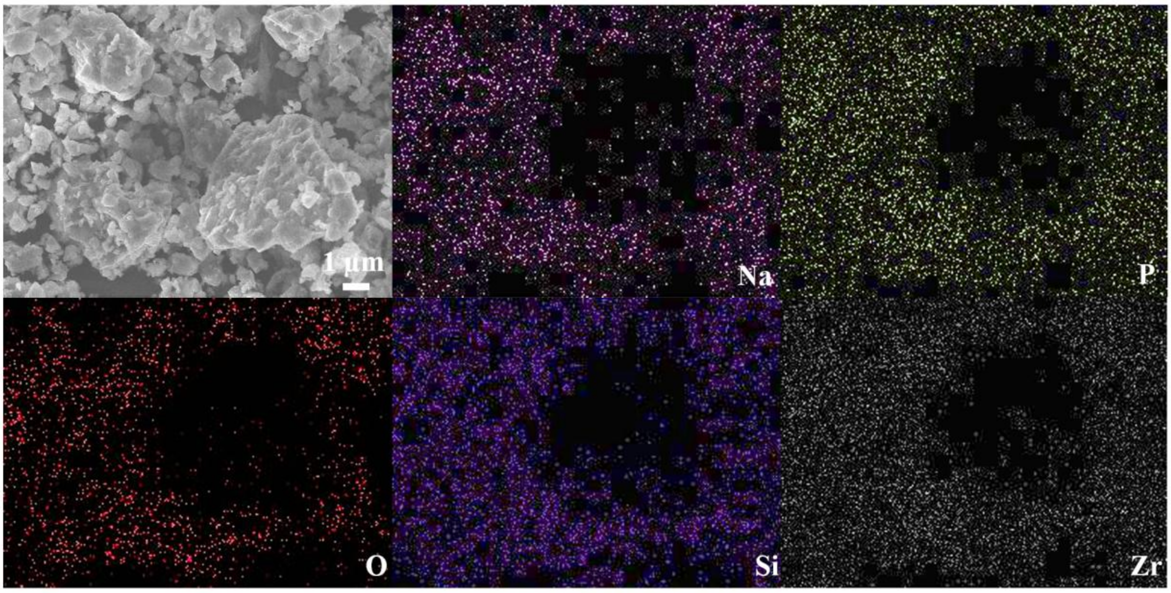

Fig. S2. SEM/EDX mapping of $Na_3Zr_2Si_2PO_{12}$ powder.

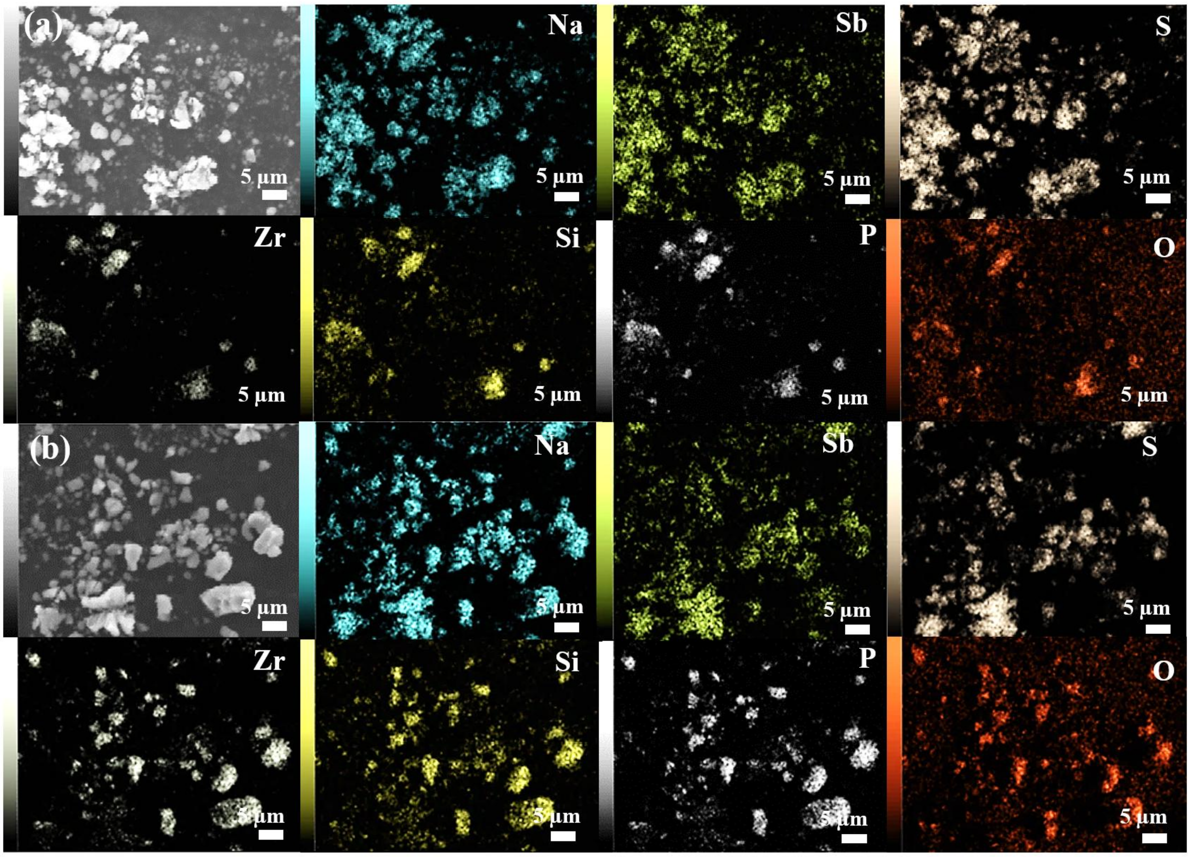

Fig. S3. SEM/EDX mapping of composite powder NSS-NZSP at (a) 70-30 wt.% and (b) 50-50. wt%, showing elemental distribution.

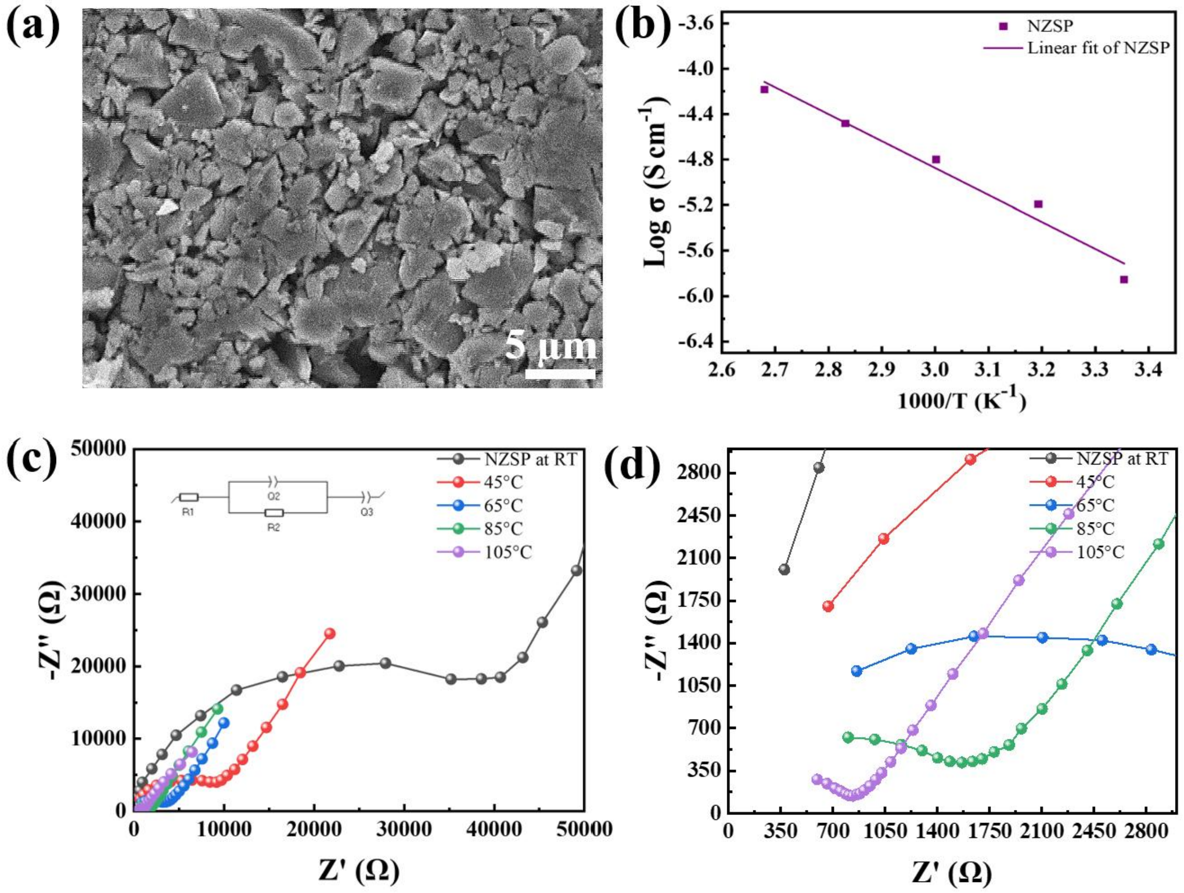

Fig. S4. (a) SEM image, (b) Arrhenius plot, and (c-d) Nyquist plots from 25 to 105 °C of commercial $Na_3Zr_2Si_2PO_{12}$ (NZSP – 360 MPa), (d) Magnified view of the high-frequency region.

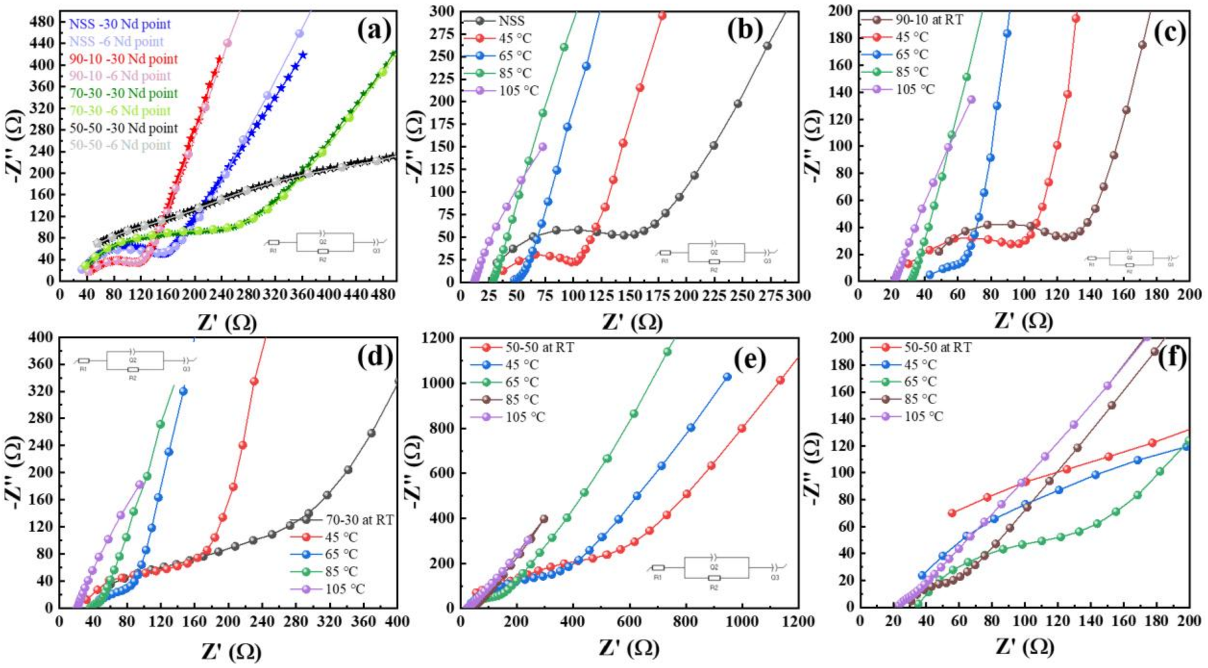

Fig. S5. Nyquist plots of NSS-NZSP composites: (a) overall composites at RT with varying node points, (b) Na_3SbS_4, (c) 90-10, (d) 70-30, (e) 50-50, and (f) high-frequency region of 50-50, measured at various temperatures from 25 to 105 °C.

Table S1. Relative density and related pellet properties of NSS, NZSP, and their composites.

Sample	Mass of pellet (*g*)	Thickness (*cm*)	Volumetric Density (*g/cm*^{3})	Relative Density (%)	$\sigma_{25\,°C}$ (mS cm^{-1})
**NSS**	0.065	0.043	1.92	66.2	0.374
**90-10**	0.073	0.042	2.21	75.2	0.397
**70-30**	0.060	0.039	2.06	68.4	0.209
**50-50**	0.070	0.040	2.28	73.8	0.087
**NZSP**	0.060	0.040	1.91	58.4	0.004

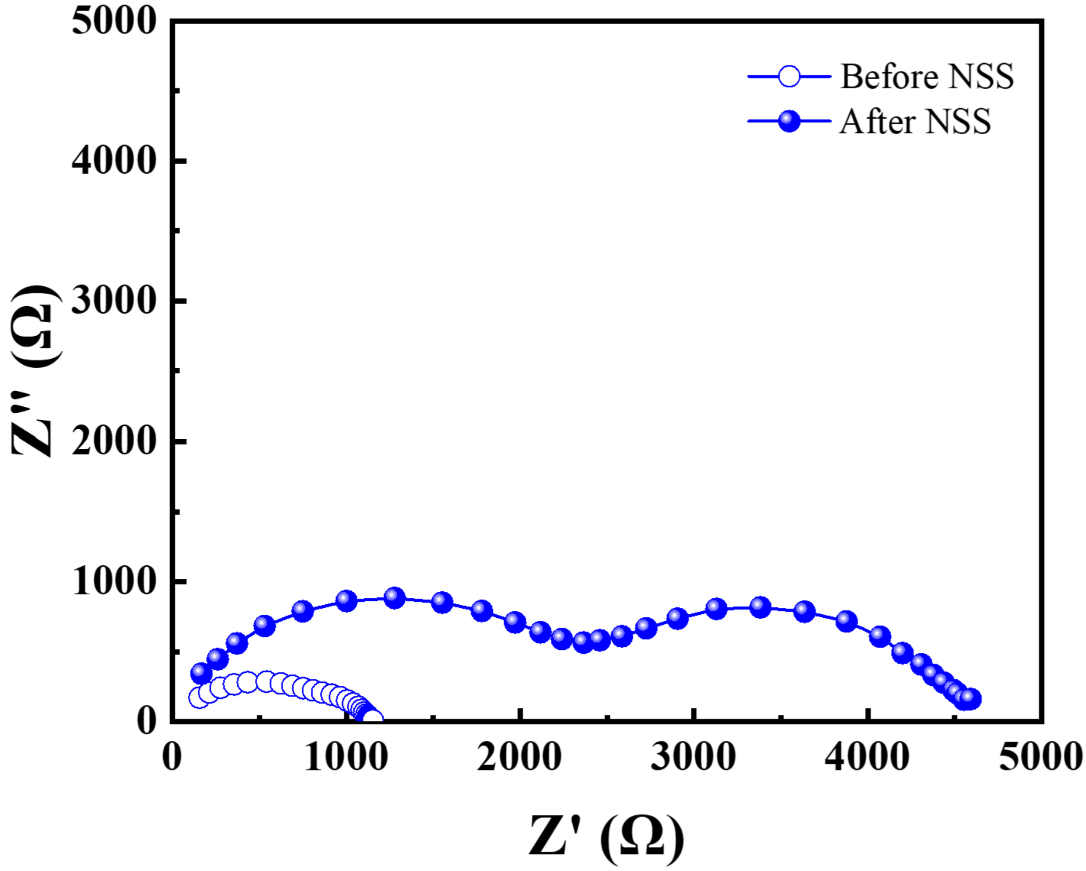

Fig. S6. Nyquist plot from DC polarization of Na_3SbS_4 solid electrolyte.

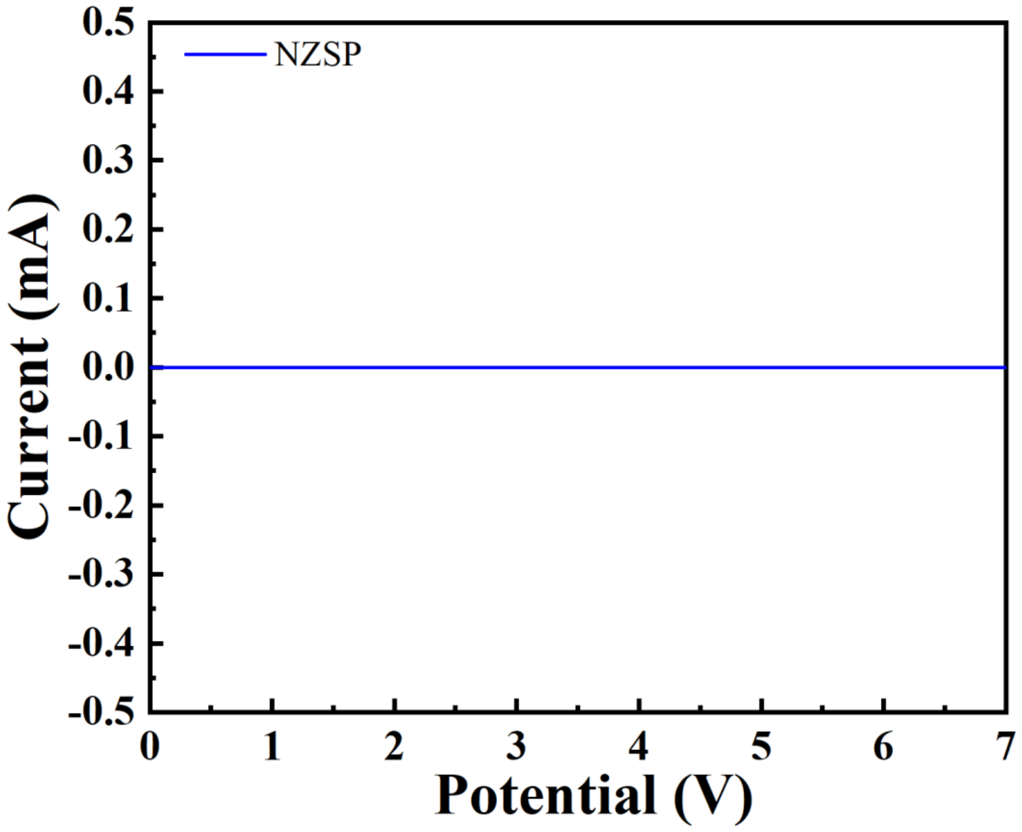

Fig. S7. LSV curve of $Na_3Zr_2Si_2PO_{12}$ at a scan rate of 0.1 mV s^{-1}.

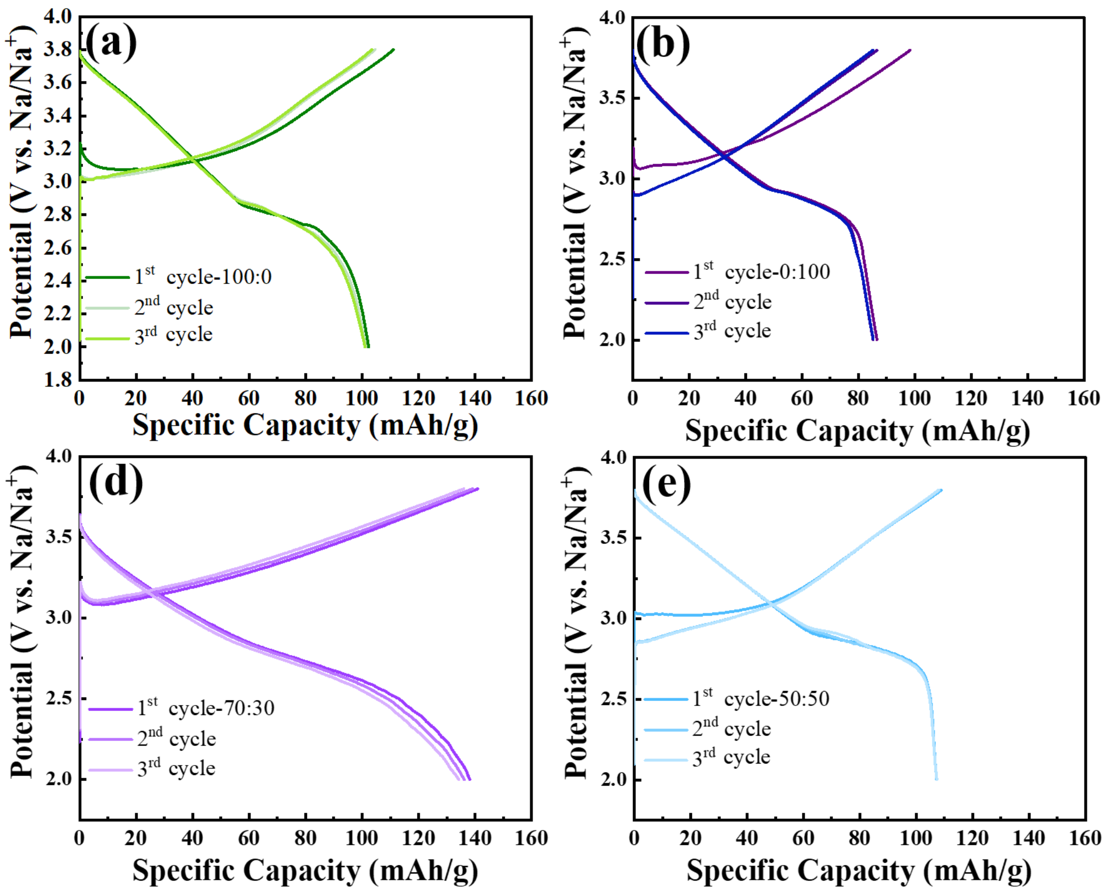

Fig. S8. Galvanostatic charge–discharge profiles (NFMO|SEs|Na) of (a) NSS, (b) NZSP, (c) 70-30 wt%, and (d) 50-50 wt.% composite NSS-NZSP solid electrolytes at 0.01 A/g at room temperature (RT).

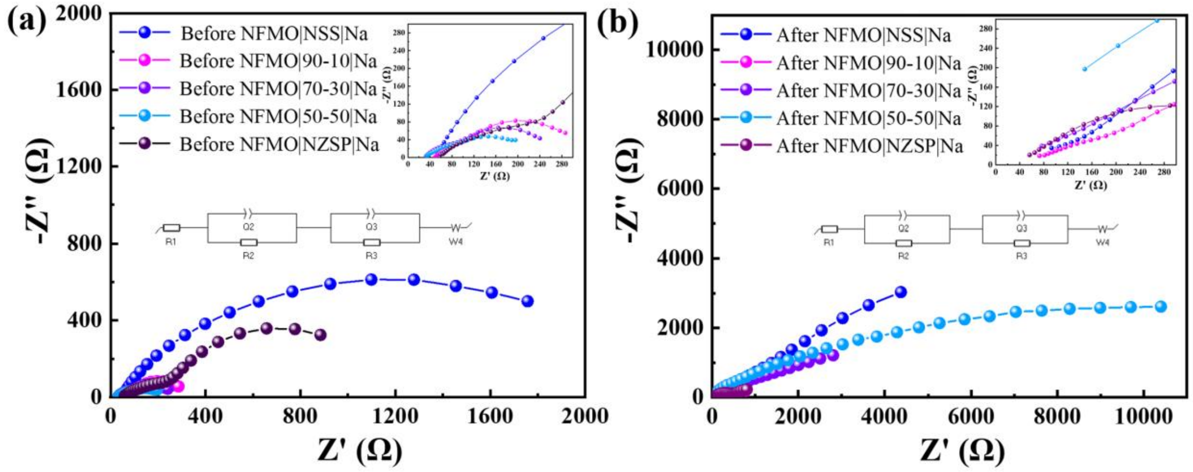

Fig. S9. (a, b) Nyquist plots of different wt.% of NZSP in NSS (a) before and (b) after 100 cycles at 0.05A/g. Insets: high-frequency region and equivalent circuit.

Table S2. Resistance values of NSS, NZSP, and composites before and after 100 cycles at 0.05 A/g current density.

**NFMO|Na at RT**	**R_1(Ω)**	**R_2(Ω)**	**R_3(Ω)**
Before NSS	50.48	443.3	1678
After NSS	68.73	1954	5835
Before 90-10	49.46	66.72	148.8
After 90-10	53.06	144.7	449
Before 70-30	32.14	63.78	142.7
After 70-30	37.46	212.9	2893
Before 50-50	31.78	51.24	111.5
After 50-50	64.48	804.6	8464
Before NZSP	54.69	236.6	769.4
After NZSP	39.69	514.2	652
